# Papillophlebitis in a COVID-19 patient: Inflammation and hypercoagulable state

**DOI:** 10.1177/1120672120947591

**Published:** 2020-07-30

**Authors:** Alfredo Insausti-García, José Alberto Reche-Sainz, Celia Ruiz-Arranz, Ángel López Vázquez, Manuel Ferro-Osuna

**Affiliations:** 1 Vitreous and Retina Unit, Department of Ophthalmology, University Hospital 12 de Octubre, Madrid, Spain; 2 Neuro-ophthalmology Unit, Department of Ophthalmology, University Hospital 12 de Octubre, Madrid, Spain; 3 Department of Ophthalmology, University Hospital 12 de Octubre, Madrid, Spain; 4 Department of Ophthalmology, University Hospital Ramón y Cajal, Madrid, Spain

**Keywords:** Venous occlusive disease, retina, retinal pathology/research, infections disease/AIDS, neuro ophthalmology, optic neuropathy, retinal degenerations associated with systemic disease

## Abstract

**Introduction:**

Papillophlebitis is a rare condition characterized by venous congestion and optic disc edema, which has been suggested to occur as a consequence of inflammation of the retinal veins or, possibly, the capillaries of the optic disc, leading to venous insufficiency and compression of the central retina vein. The disease affects healthy young adults and commonly has a benign course, however, if complications such as macular edema or ischemia appears, treatment should be instituted immediately to avoid poor prognosis.

**Case report:**

A 40-year old white male patient consulted for a slight decrease in the sensitivity of the visual field in his left eye (OS). Visual acuities (VA) were 20/20 in both eyes. OS fundus examination showed dilated and tortuous retinal vessels, disc edema, and retinal hemorrhages. The patient was diagnosed with papillophlebitis. OS VA decreased to 20/200 due to macular edema, and he was treated with a intravitreal dexamethasone implant. An exhaustive and interdisciplinary exploration process was performed, identifying a recent disease and recovery of Covid-19 as the only factor of inflammation and coagulation alteration. Other systemic diseases were excluded. We also describe a rapid decrease in disc and macular edema after intravitreal dexametasone injection, which could support the inflammatory hypothesis.

**Conclusion:**

The importance of this case lies in the possible association of papillophlebitis with the new Covid-19 disease. We believe that the inflammatory reaction and the coagulation alteration present in our patient due to Sars-Cov2 coronavirus may have acted as risk factors for the development of papillophlebitis.

## Introduction

Since December 2019, coronavirus disease 2019 (COVID-19) has become a global pandemic caused by the novel and highly transmissible enveloped RNA virus named severe acute respiratory syndrome coronavirus 2 (SARS-CoV-2), identified as a member of the Coronaviridae family.^1^

The complete spectrum of clinical manifestations associated with COVID-19 is not fully elucidated, as far as new clinical symptoms are often described. To this day clinical presentations range from minor unspecific symptoms, such as fever, anosmia, ageusia, dry cough or diarrhea, to severe pneumonia and disturbed gas exchange leading in approximately 3% to 10% of infected patients to acute respiratory distress syndrome, multi-organ failure, septic shock, or even death.^2,3^

Papillophlebitis is an uncommon disease considered as a clinical variant of central retinal vein occlusion (CRVO) that is typically seen in healthy young adults. It has been suggested to result from idiopathic inflammation of retinal vascular and, possibly of the capillaries of the optic disc; however it is mandatory to work out a hypercoagulable state (hereditary or acquired thrombophilia factors), vasculitic syndromes, blood hyperviscosity, and other recognized systemic vascular inflammatory disorders.^4^ The most frequently reported visual symptoms are a slight and painless unilateral VA decrease, often inconspicuous on physical examination, and visual field testing commonly shows an enlarged blind spot. Unlike typical CRVO, patients with papillophlebitis usually present with normal or near normal VA, and visual final prognosis is more favorable.^4,5^

## Case description

A 40-year-old white male with the main complaint of persistent and painless decrease in the sensitivity of his vision in his left eye (OS) was evaluated. VA was 20/20 in both eyes. Anterior segment examination and intraocular pressure were not remarkable. There was no pain on ocular movements and there was no relative afferent pupillary defect (RAPD).

On left eye fundus examination, and color and red free retinographies, severe inflammation of the optic nerve head was observed accompanied by retinal venous vasodilatation and tortuosity, cotton-wool spots and moderate superficial hemorrhages in all four quadrants. Fluorescein angiography (FA) performed in the acute phase of the clinical presentation showed a discrete venous staining and leakage, in addition to leakage and late staining from the optic disc. There was no evidence of areas of ischemia or peripheral vasculitis.

**Figure 1.**
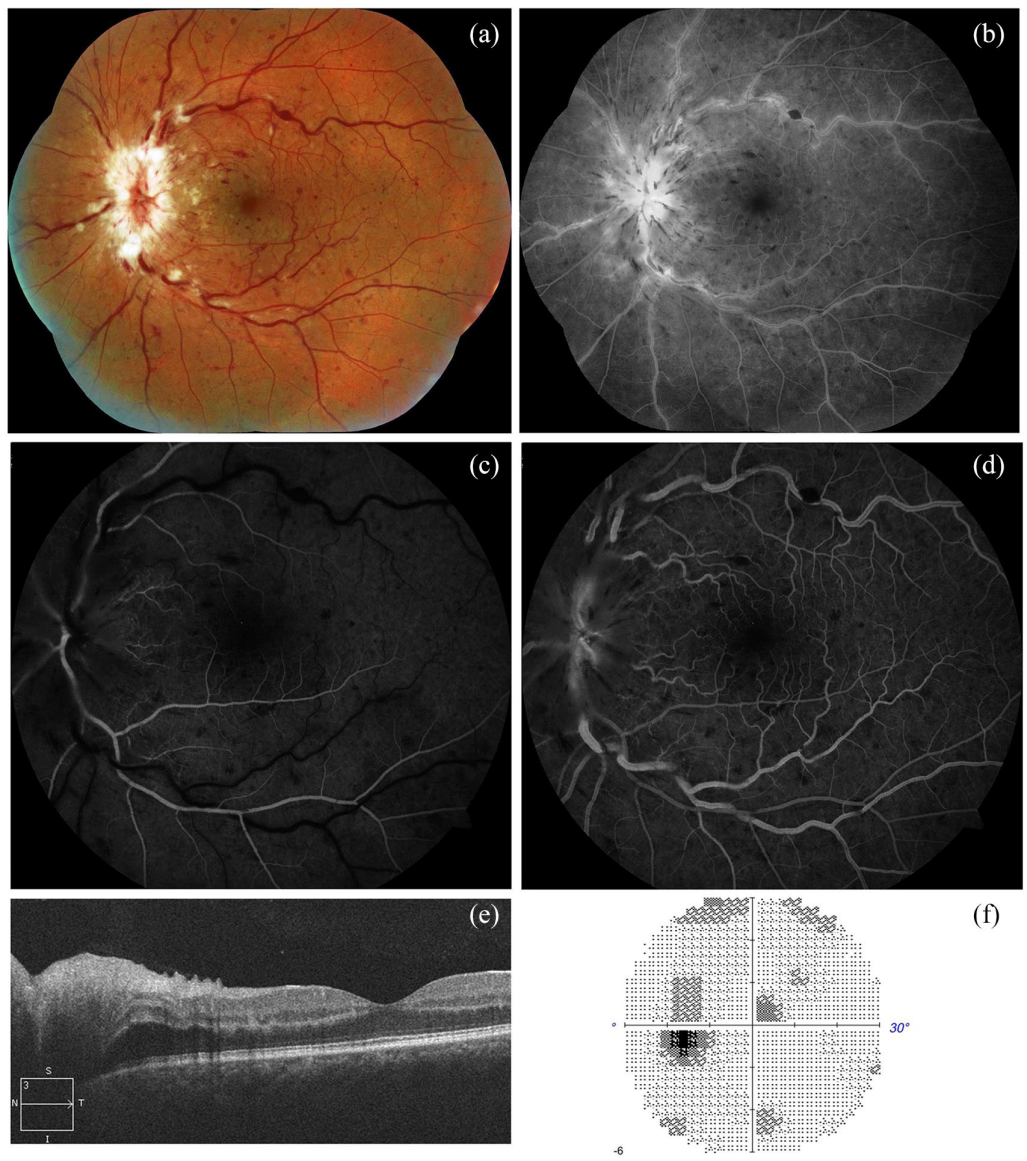
(a) Retinography and (b) red free retinography: inflammation of the optic disc, retinal venous vasodilatation and tortuosity, and superficial hemorrhages in all four quadrants. (c) Early arteriovenous phase FA and (d) arteriovenous phase FA: discrete venous staining and leakage, in addition to leakage and late staining from the optic disc. No evidence of areas of ischemia or peripheral vasculitis. (e) OCT: optic disc edema without macular edema. (f) VF: slight central scotoma and a slight to moderate increase in the blind spot.

In the visual field, a diffuse sensitivity decrease was observed, associated with a slight central scotoma and a moderate increase in the blind spot. Initial optical coherence tomography (OCT) showed papillary edema without evidence of involvement of the macular area (Figure 1). The patient was diagnosed with papillophlebitis.

The patient indicated that 6 weeks before the onset of visual symptoms, he had presented high fever, persistent cough, and myalgia for approximately 2 weeks, at the beginning of the coronavirus pandemic in Madrid (Spain). The patient was followed up on an outpatient basis and without the need for systemic treatment. At that time, the patient did not undergo a polymerase chain reaction (PCR) diagnostic test for SARS-CoV-2. Blood pressure was normal, and patient did not report other relevant medical conditions or previous eye history.

Treatment was started with acetylsalicylic acid orally in the dose of 100 mg once a day and bromfenac 0.9 mg/mL eye drops one drop twice daily, awaiting the results of the studies and tests requested.

No pathological findings were observed in requested blood analysis comprising complete blood count, glycemia, lipidic profile, homocysteinemia, anti-cardiolipin IgM and IgG antibodies, and screening for genetic thrombophilia (Factor V Leiden and prothrombin mutations, antithrombin III and proteins C and S deficiencies). Also prothrombin time (PT) and activated partial thromboplastin time (APTT) were normal. Chest X-ray and brain magnetic resonance were unremarkable. The patient was positive for serum IgM and IgG SARS-CoV-2 qualitative enzyme-linked immunoassay (ELISA; positive value >1.0) and showed persistent alteration of parameters of the coagulation system: D-dimers (672 μg/L, normal value: <460 μg/L), fibrinogen (451 mg/dL, normal value: 200–400 mg/dL). The C-reactive protein (CRP) value at diagnosis was 0.898 mg/dL, normal value: <0.500.

**Figure 2.**
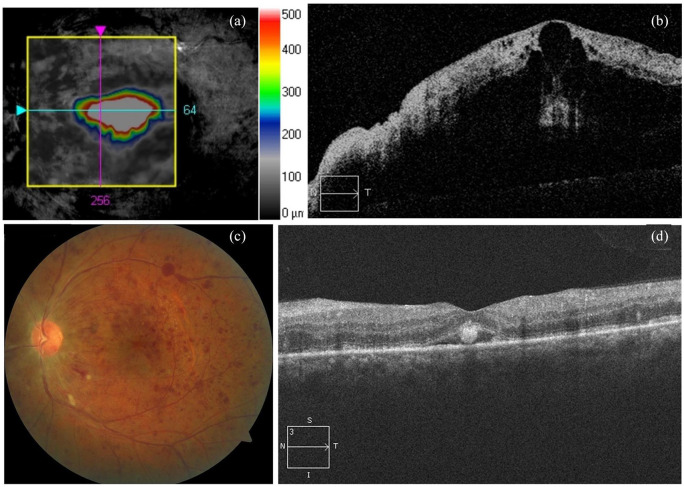
(a and b) Macular optical coherence tomography (OCT) demonstrating cystoid macular edema (CMO). (c) Retinography and (d) macular OCT: 2 weeks after intravitreal dexamethasone sustained-release implant treatment. Marked improvement of vascular tortuosity, optic disc, and macular edema.

One week after diagnosis, patient visual acuity decreased to 20/200, due to macular edema. Sustained-release dexamethasone implant (Ozurdex, Allergan, Dublin, Ireland) was intravitreal injected, showing a marked decrease in macular and papillary edema and a progressive and gradual recovery of vision as of 20/40 2 weeks later (Figure 2). Written informed consent was obtained for the publication of this case report.

## Discussion

The ocular implications of SARS-CoV-2 in humans have not been widely identified. Previous reports include conjunctival hyperemia, chemosis, epiphora, and increased secretions mostly reported during the middle phase of the disease, and in patients with pneumonia.^6^ In retina, subtle cotton wool spots and micro-hemorrhages at the fundus examination and hyper-reflective lesions at the level of ganglion cell and inner plexiform layer at the papillomacular detected by OCT, have been described.^7^ More severe ocular manifestations as anterior uveitis, retinitis, and optic neuritis have been only documented in animal models.^8^

In addition to the respiratory tract infection and to these acute ocular manifestations, the current pandemic caused by the SARS-CoV-2 is associated with coagulation activation and a disproportionate systemic inflammatory response.^1,3,9^ The available information suggests that SARS-CoV-2 binds to host cells via the angiotensin converting-enzyme (ACE) 2 receptor (R) – a metallopeptidase. These receptors are present in all major organs, however, the density is particularly high in the lungs, heart, veins, and arteries. The high expression of ACE2 receptors within endothelial cells raises a question of its vulnerability to SARS-CoV-2 binding, and the pathogen’s ability to produce systemic endothelial dysfunction.^9^ Endothelial alterations and endotheliitis are important determinants of microvascular dysfunction, since it displaces the vascular balance toward vasoconstriction, ischemia, tissue edema, and a procoagulant state. Parallel to the alteration of coagulation, an exacerbated proinflammatory cytokines response has been observed in Covid-19 patients (CRP, Ferritin, IL-2, IL6, IL-7, IL-10, IP-10, TNF-α, etc.), although the cause of this cytokine storm is not yet clear. The SARS-CoV-2 cytokine storm precipitates the onset of a systemic inflammatory response syndrome, resulting in the activation of the coagulation cascade that induces to a hypercoagulable state. However, whether the coagulation cascade is directly activated by the virus or whether this is the result of local or systemic inflammation is not completely understood. These findings are consistent with the close connection between thrombosis and inflammation.^9^

For these reasons it is believed that many patients with COVID-19 may have thrombotic microangiopathy and venous or arterial thromboembolic complications. Some studies have shown an incidence of venous and arterial thrombotic events in more than 30% of COVID-19 patients, with venous thromboembolic events being the most common (27%).^9,10^ Patients with moderate to severe infection are more likely to have COVID-19-associated coagulopathy (CAC) and these thrombotic complications can occur even in late stages of the disease or early recovery. The most common pattern of coagulopathy observed in patients hospitalized with COVID-19 is characterized by elevations in fibrinogen and D-dimer levels. This correlates with a parallel rise in markers of inflammation.^9^

Papillophlebitis is considered a subset of CRVO that occurs in young and healthy patients.^4^ Due to its presentation and clinical evolution, it is a different disorder from the typical CRVO in which venous thrombosis derived from the compression of an atherosclerotic central retinal artery over the central vein.^4,5^ Fundus examination is characterized by unilateral blurred borders of the optic head nerve, swelling and hyperemia, retinal venous tortuosity and vasodilation, cotton wool spots, and a variable extent of intraretinal hemorrhage, associated or not to macular edema. Ophthalmological examination does not indicate an optic nerve conduction defect and RAPD is typically absent. At the time of diagnosis, macular OCT is usually normal.

The differential diagnosis of papillophlebitis includes CRVO, nonarteritic ischemic optic neuropathy, diabetic papillopathy, infectious papillitis, hypertensive retinopathy, prethrombotic retinal vein, and orbital compressive lesions. The clinical manifestation, the age of presentation, the absence of an afferent pupillary defect, the absence of cardiovascular risk factors and the performance of complementary studies will make it possible to differentiate these diseases from each other. An evaluation for hypercoagulable disorders must be considered. In this patient, an interdisciplinary exploration process and a complete thrombophilia study was performed, and the only findings were consistent with a hypercoagulable state induced by the SARS-CoV-2 infection.

Usually the clinical course of papillophlebitis is typically without permanent visual loss, and spontaneous improvement is common. However, the condition is not always benign, up to 30% of patients with papillophlebitis develop ischemic venous occlusion and could develop neovascular glaucoma; and some will have macular edema leading to poor visual acuity. Consequently frequent ophthalmologic examinations are required in patients with papillophlebitis.

Decreased VA secondary to macular edema or areas of ischemia and neovascularization are an indication to establish treatment. If they are not diagnosed and treated immediately, papillophlebitis may will have a poor prognosis.

## Conclusion

We currently know that patients affected by COVID-19 are at risk of presenting venous and arterial thrombotic events, and that these thrombotic events are actively involved in the pathophysiology of the disease. The three main factors involved in the pathogenesis of coagulopathy in patients with COVID-19 are: endotheliitis, which causes mechanical problems through vasoconstriction, the phenomenon of hyper-inflammation and cytokine storm which activates clotting factors, and lastly, stasis, and hypoxia, that also activates coagulation mechanisms. Ophthalmologists must be prepared and aware that beyond the involvement of the ocular surface that occurs in the intermediate stages of COVID-19 for which we can be consulted; there may be an increase in the incidence of patients with ocular vascular diseases due to the hyperinflammatory and hypercoagulable state triggered by infection of SARS-CoV-2.
